# SARS-CoV-2 Codon Usage Bias Downregulates Host Expressed Genes With Similar Codon Usage

**DOI:** 10.3389/fcell.2020.00831

**Published:** 2020-08-20

**Authors:** Andres Mariano Alonso, Luis Diambra

**Affiliations:** ^1^InTech, Universidad Nacional de San Martin, Chascomús, Argentina; ^2^CONICET, Chascomús, Argentina; ^3^CREG, Universidad Nacional de La Plata, La Plata, Argentina

**Keywords:** SARS-CoV-2, codon usage bias, codon optimality, translational control, pathogeny, vaccine design

## Abstract

Severe acute respiratory syndrome has spread quickly throughout the world and was declared a pandemic by the World Health Organization (WHO). The pathogenic agent is a new coronavirus (SARS-CoV-2) that infects pulmonary cells with great effectiveness. In this study we focus on the codon composition for the viral protein synthesis and its relationship with the protein synthesis of the host. Our analysis reveals that SARS-CoV-2 preferred codons have poor representation of G or C nucleotides in the third position, a characteristic which could result in an unbalance in the tRNAs pools of the infected cells with serious implications in host protein synthesis. By integrating this observation with proteomic data from infected cells, we observe a reduced translation rate of host proteins associated with highly expressed genes and that they share the codon usage bias of the virus. The functional analysis of these genes suggests that this mechanism of epistasis can contribute to understanding how this virus evades the immune response and the etiology of some deleterious collateral effect as a result of the viral replication. In this manner, our finding contributes to the understanding of the SARS-CoV-2 pathogeny and could be useful for the design of a vaccine based on the live attenuated strategy.

## 1. Introduction

The new SARS-CoV-2 coronavirus is the causative agent of the current pandemic of COVID-19. This highly pathogenic virus has quickly become the latest threat to modern life. From the end of 2019 and up to the redaction of this paper, this virus has infected over 17 million people, leading to mild symptoms of fever and to lung function reduction, severe acute respiratory syndrome (SARS), and even death. The current global death toll stands at 670,000; however, we are still far away from determining the final mortality figure. In the absence of vaccines or effective antiviral treatments against SARS-CoV-2, it is important to understand how this virus appropriates the host translation apparatus and subverts the immune defenses of infected cells. This can be the first step in the development of novel therapeutics.

As intracellular parasites, virus replication depends on the translational machinery of their cellular hosts to translate viral transcripts. Thus, virus replication requires ribosomes, tRNA, and translation factors from the cell host. On the other hand, codon usage bias is a feature of natural selection and affects the genomes of all domains of life. It is known that more frequently used codons are used for coding highly abundant proteins (Pan et al., 1998; Dana and Tuller, 2014; Quax et al., 2015; Diambra, 2017). Virus genomes also have preferences in the codon usage, but, in this case, the bias is constrained by the host translational machinery (Shackelton et al., 2006). The effects of codon composition of a transcript on its translation have been reported in literature (Gingold and Pilpel, 2011; Plotkin and Kudla, 2011; Shah and Gilchrist, 2011; McCarthy et al., 2017) and is considered an important determinant of gene expression (Zhou et al., 2009; Tuller et al., 2010a,b). However, the codon usage of a gene can also affect the translation of other genes (Frumkin et al., 2018). In fact, the virus replication demands not only ribosomes but also a lot tRNA resources for the codons highest in demand (Chen et al., 2020). The consumption of specific tRNAs for the virus replication could thus be an alternative to controlling host protein synthesis machinery as well as generating deleterious collateral effects on the function of the host cells. Recent evidence supports the idea that high codon usage similarity between virus and host can lead to a deleterious effect on the host (Chen et al., 2020). Furthermore, Chen et al. (2020) have shown that codon composition of highly expressed viral genes regulates the tRNA availability, affecting the decoding time of codons in the infected cells. In this manner the viral infection can convert abundant codons into scarce codons, reshaping the human codon optimality pattern. In this sense, it has been proposed that dysregulation of the tRNA pool can lead to disease (Dhindsa et al., 2020). In fact, there is growing evidence supporting that synonymous variations in a coding sequence can affect its folding and/or resulting expression level (Tsai et al., 2008; Hunt et al., 2014; Buhr et al., 2016; Kirchner et al., 2017; von Herrmann et al., 2018; Dhindsa et al., 2020; Kim et al., 2020).

It is known that coronavirus genomes are poor in GC content (~40%) and that there exist a preferential use of A-ended or U-ended codons in these genomes (Gu et al., 2004; Kandeel et al., 2020). The last bias is usually characterized in the literature as the GC3 content. Indeed, the analysis performed by Gu et al. (2004) suggests that this compositional constraint is a major determinant to synonymous codon usage. Here, we study recent proteomic data from SARS-CoV-2 infected cells (Bojkova et al., 2020) from a novel point of view. By considering the codon usage of the virus ORFeome, we characterize a set of genes whose expression could be affected by the massive demand of the tRNA which implies the virus replication. We find that those host genes encoding proteins with similar codons to the virus ORFeome have a lower translational rate. Extrapolating this finding to highly expressed genes in lung, we find a small set of genes that can be downregulated. These genes are involved in translation, immune systems, and cell calcification, to name a few, and their roles in the SARS-CoV-2 cell pathogenesis should be the target of further studies.

## 2. Materials and Methods

The coding sequences associated with the genome of SARS-CoV and SARS-CoV-2 were obtained from the NCBI (NC_004718.3 and NC_045512.2, respectively). Highly expressed genes in the lung and arterial tissues were retrieved from the GTEx portal (gtexportal.org). Proteomic and translatomic data from infected CACO-2 cells were retrieved from Supplementary Material of Bojkova et al. (2020), which are publicly available (ProteomeXchange repository, ID = PXD017710). These data contain protein levels from 6,381 proteins and translational rate from 2,715 proteins, and the proteomic information is consequently not available for many proteins.

Given the coding sequence *s*, we compute the codon usage frequency as $f_{s}\left(c\right)=\frac{N_{c}}{L_{s}}$, where *L*_*s*_ is total number of codons in the sequence *s*, and *N*_*c*_ is the number of times that codon *c* is present in *s*. In a similar manner, we define codon usage frequency relative to the viral ORFome: $f_{O}\left(c\right)=\frac{N_{c}}{L_{O}}$, where *L*_*O*_ is the total number of codons in the ORFome, and *N*_*c*_ is the number of occurrences observed codon *c* in the ORFome. Thus, *f*_*s*_(*c*) and *f*_*O*_(*c*) are vector of 64 elements. To compute the CCorr of a given sequence *s* with viral ORFome, we consider the Pearson's correlation coefficient between the vector *f*_*s*_(*c*) and *f*_*O*_(*c*):

(1) $$\begin{array}{l} \text{CCorr}=\frac{\sum_{\text{c}}\left({\text{f}}_{\text{s}}\left(\text{c}\right)-\bar{{\text{f}}_{\text{s}}}\right).\left({\text{f}}_{\text{O}}\left(\text{c}\right)-\bar{{\text{f}}_{\text{O}}}\right)}{\sqrt{\sum_{\text{c}}{\left({\text{f}}_{\text{s}}\left(\text{c}\right)-\bar{{\text{f}}_{\text{s}}}\right)}^{2}}.\sqrt{\sum_{\text{c}}{\left({\text{f}}_{\text{O}}\left(\text{c}\right)-\bar{{\text{f}}_{\text{O}}}\right)}^{2}}} \end{array}$$

## 3. Results

**Figure 1:**
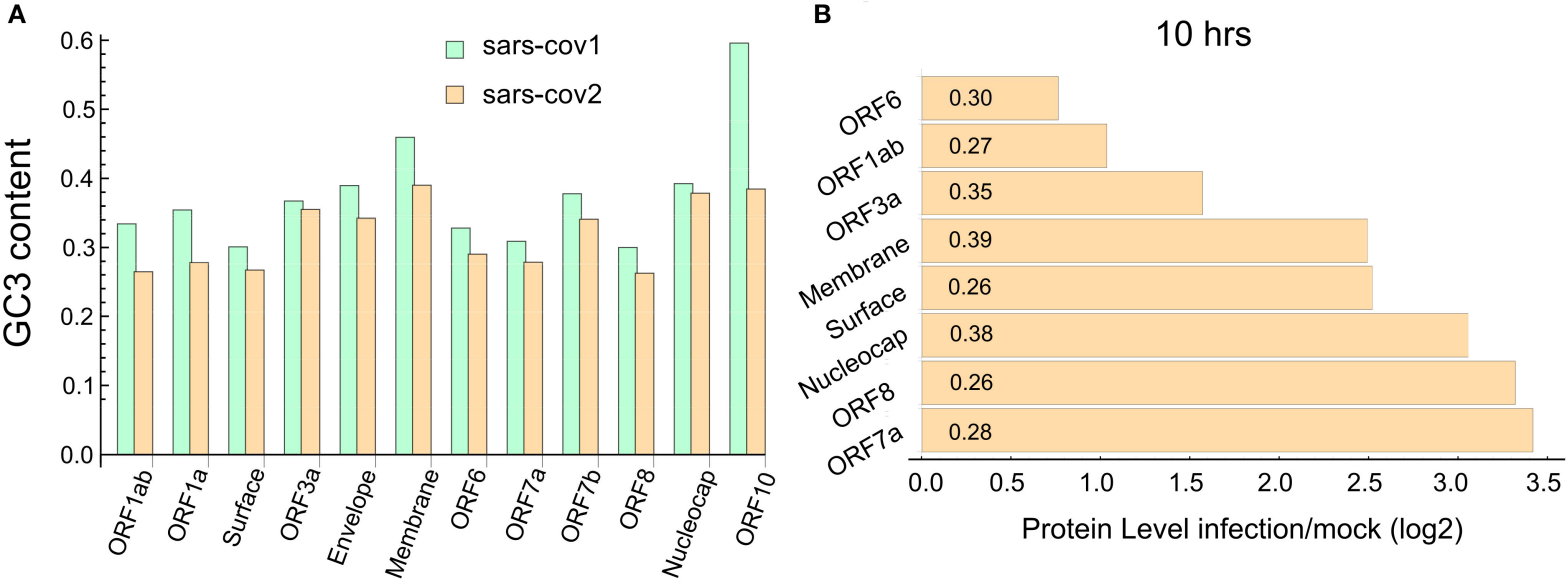
SARS-CoV-2 ORFome have lower content of GC3. **(A)** Bar chart comparing the G and C nucleotides on the third nucleotide (GC3) at every codon in the ORFeome of SARS-CoV and SARS-CoV-2. **(B)** Bar chart showing the proteins level of nine viral proteins at 10 h post-infection (data obtained from Bojkova et al., 2020). The GC3 content for each viral ORF is showed in the corresponding bar.

We study the codon composition of two complete coronavirus genomes by measuring the percentage of G and C nucleotides at the wobble position of the codons (GC3 content). Figure 1A depicts the GC3 content of each annotated coding sequence of the SARS-CoV and SARS-CoV-2. This analysis reveals that, in agreement with Gu et al. (2004) and Kandeel et al. (2020), codons preferentially used by these viruses are associated with a lower GC3 content than that expected from the random use of nucleotides. In particular, the ORFs corresponding to the proteins ORF1ab, ORF1a, surface, ORF6, ORF7a, and ORF8 exhibit a very low content of GC3. Specifically, the last two proteins are highly expressed at 10 h post-infection (PI), as can be seen in Figure 1B.

**Figure 2:**
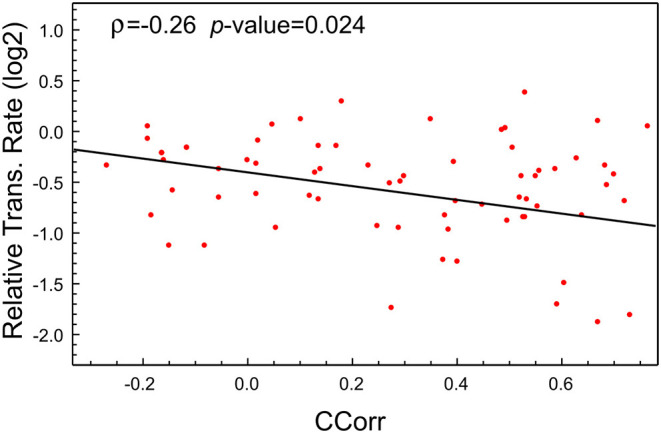
The translational rate of genes with high CCorr in SARS-CoV-2-infected cells is lower than the mock-infected cells. Scatter plot showing negative correlation between translational rate of the 100 most expressed proteins in SARS-CoV-2 infected CACO-2 cells vs. virus codon usage.

As was highlighted previously, this high expression of viral proteins could lead to an imbalance in the tRNA pool needed for the normal synthesis of the proteins of the host cell. Of course, as tRNA pools depend on the cell types, the postulated imbalance could be a tissue-dependent feature. To check this hypothesis, we make use of the recently available data about the proteome profile and translational rate in SARS-CoV-2-infected cells (Bojkova et al., 2020). In this study, a CACO-2 cell line was infected with SARS-CoV-2 and mocked, and the last one was used as the control. From this proteome, we selected the coding sequences corresponding to the 100 most abundant proteins in the mock infected cells at 10 h PI. We then computed the codon correlation (CCorr) between the codon usage of each one of these sequences and the codon usage of all coding sequences in SARS-CoV-2. Figure 2 depicts a scatter plot of the translational rate of these genes from SARS-CoV-2-infected cells vs. the corresponding CCorr (red dots). The black line is the adjusted linear model, which shows a significant and negative correlation between the translation rate and the codon composition of each sequence. This means that the translational rate of coding sequence in SARS-CoV-2-infected cells is lower than in mock-infected cells. Although the correlation (ρ = −0.26) with codon composition is not high, reflecting the fact that other factors could be regulating the translation rate, it is significant at a value of 0.05 (*p*-value = 0.024 < 0.05).

**Figure 3:**
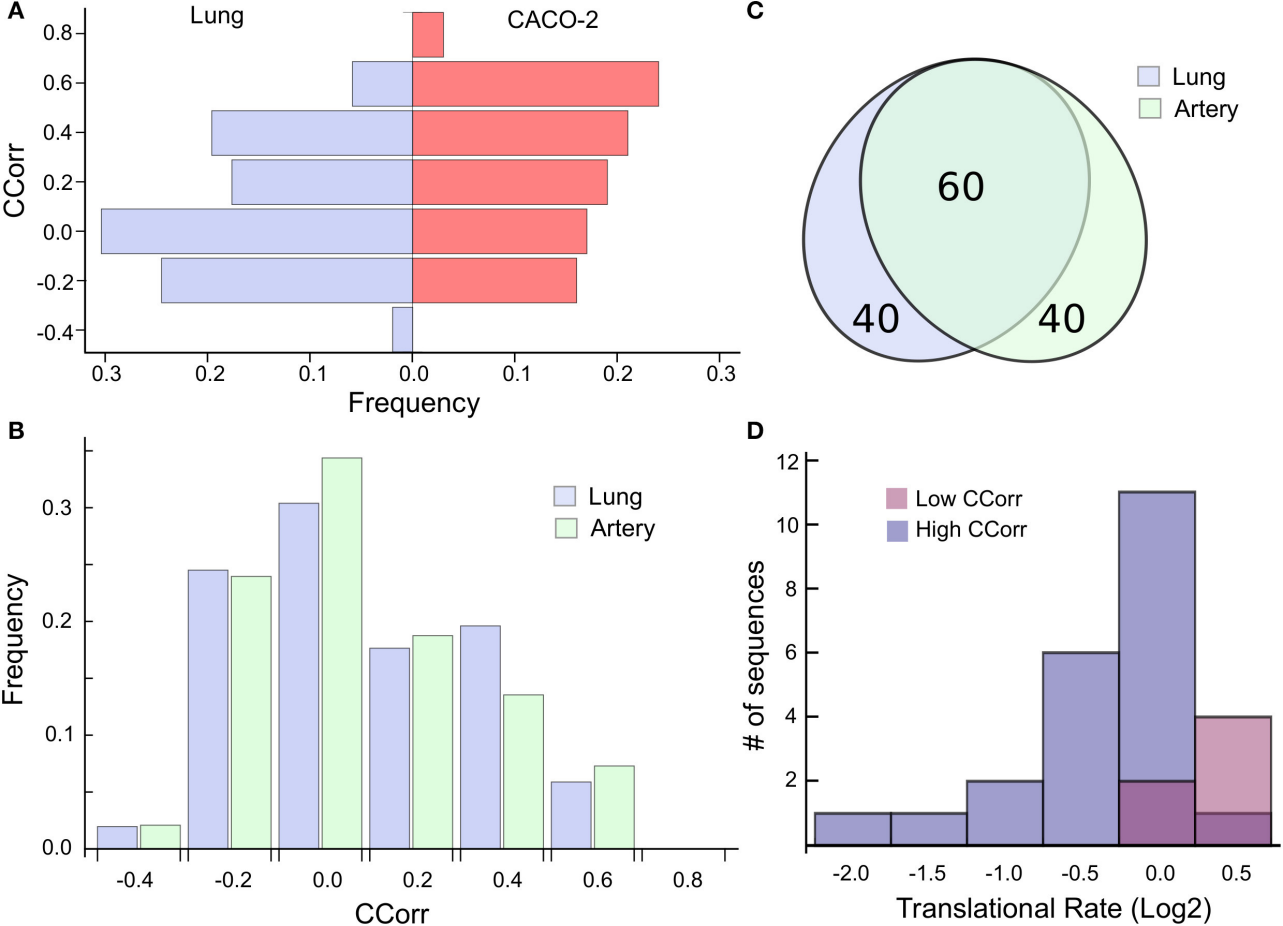
High codon correlation between SARS-CoV-2 and lung cells is associated with a translational rate decay over specific transcripts. **(A)** Frequency distribution of CCorr on the 100 highest expressed genes in lung cells and CACO-2 cells. **(B)** Comparison between the CCorr frequency distributions obtained from the 100 highest expressed genes in lung and arterial tissues. **(C)** The tissues of the lungs and arteries share most of the highly expressed genes. **(D)** Histogram of the translational rate of 100 highest expressed genes in lung cells. Transcripts with high and low codon correlation (CCorr) with SARS-CoV-2 are highlighted.

This analysis suggests then that the codon composition of highly expressed host-genes is a determinant of its own translation rate and that viral replication induces a particular case of epistasis, which could affect host cell proteostasis. Consequently, we find it relevant to extrapolate this phenomenon to cells affected by SARS-CoV-2. In this sense, the ACE2 receptor has been identified as the SARS-CoV-2 cell entry. Even though ACE2 transcript is present in almost all organs, the surface expression of ACE2 protein is present in lung alveolar epithelial cells and arterial and venous endothelial cells (Hamming et al., 2004). For that reason, we elected to analyze the most expressed genes in lung and arterial tissues extracted from the GTEx database, two main targets of the SARS-CoV-2 infection. To this end, we select the 100 most expressed genes in these tissues and compute the CCorr for each sequence. Figure 3A shows that the codon composition profile of the most expressed genes from both lung and CACO-2 cells types are different, it is evident that the lung cells share fewer codons in common with the SARS-CoV-2 than the CACO-2 cells. On the other hand, the codon composition profile of the highly expressed genes in arterial tissue are quite similar to the genes associated with the lung (Figure 3B). In fact, they share 60% of these highly expressed genes (Figure 3C). Consequently, the tRNAs pool used for the virus could be scarcer both in lung and arterial cells than in the CACO-2 cells.

We search for those genes, highly expressed in the lung tissue, which could be affected by the depletion of the tRNAs consumed by virus replication. They are those whose codon composition is similar to SARS-CoV-2 (CCorr > 0.25); these are listed in Table 1. Before analyzing these 27 genes of interest, we compared the translation rate of these genes with the translation rate associated with highly expressed genes, but which differs in their codon usage bias (CCorr < −0.075). In this sense, we used the Mann-Whitney *U* test for median differences of independent samples to analyze the difference in translational rate between these two groups of genes. This test identified significant differences between these groups (*p*-value = 0.0007), as it can be seen in Figure 3D. We want to remark that, since there is still no available data on translational rate from lung cells, the last analysis was performed by using translational rate data available from CACO-2 cells, although we know that this data would be underestimating the difference between these two groups of genes.

**Table 1:** Set of most expressed genes in lung (GTEx) and positive correlation with virus codon usage.

**Gene name**	**Codon corr**	**T. rate 6 h**	**T. rate 10 h**
RPS3A	0.582	0.686	0.980
RPL5	0.559	0.585	0.271
RPL9	0.546	0.723	0.738
SPARCL1	0.536	NA	NA
EEF1A1	0.531	0.948	0.627
RPS12	0.443	0.155	0.649
RPL17	0.438	NA	0.372
TPT1	0.415	1.048	0.981
RPL21	0.411	NA	NA
RPS6	0.398	1.206	0.590
RPL34	0.376	0.981	0.727
RPS13	0.372	1.386	0.209
RPL4	0.370	0.919	0.799
RPS27A	0.365	NA	NA
RPSA	0.352	0.902	0.643
SAT1	0.350	NA	NA
A2M	0.340	NA	NA
TXNIP	0.317	NA	NA
RPS25	0.315	0.728	0.753
FN1	0.308	NA	NA
RPL24	0.306	0.576	0.890
RPLP1	0.303	NA	NA
RPS7	0.297	0.965	1.036
HSP90AB1	0.296	0.989	0.738
B2M	0.277	0.926	0.825
S100A11	0.273	1.620	0.520
MGP	0.272	NA	NA

*col 1: gene name, col 2: CCorr, cols 3 and 4: translational rate, infected CACO-2 cells, measured at 6 and 10 h PI, respectively. Translational rates are relative to mock cells*.

**Figure 4:**
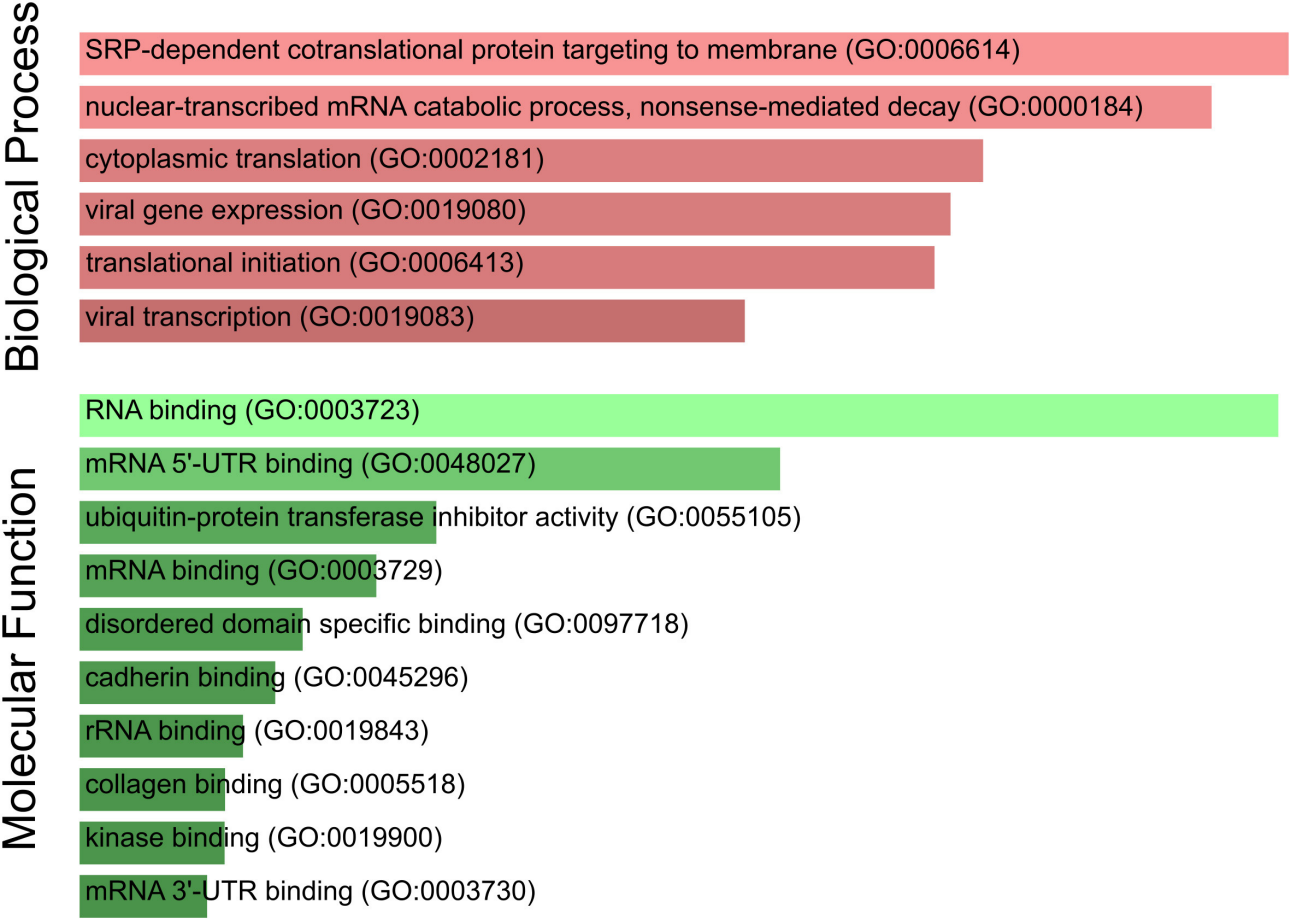
SARS-CoV-2 codon usage may have an impact on host translational machinery. GO enrichment analysis performed over genes of interest. The most enriched biological processes and molecular functions are highlighted. Both size and color shade of bars represent the level of significance.

Furthermore, we performed a GO term enrichment analysis over the 27 genes listed in Table 1; their translation rates are decreased by means of the Enrichr online software (Chen et al., 2013). Figure 4 illustrates the main enrichment pathways. This analysis reveals that codon usage could promote extensive changes in the translation machinery of the host in agreement with previous report in CACO-2 cells (Bojkova et al., 2020). It is known that when canonical translation is impaired, as part of the host defense program, specific 40S ribosomal subunits are needed to support uncapped viral mRNA translation (Kwan and Thompson, 2019).

Our list of genes from lung is enriched with several ribosomal proteins that constitute both 40S (RPS6, RPS3A, RPS7, RPSA, RPS25, RPS13, RPS12, and RPS27A) and 60S (RPL4, RPL5, RPL21, RPLP1, RPL34, RPL9, RPL24, and RPL17) ribosomal subunits. Many of them also appear in the set of genes derived from arterial tissue. All of them belong to the nonsense-mediated decay pathway that control the mRNAs with abnormal termination. The termination codon can be recognized if the 3′ untranslated region is short or if it does not have an exon junction complex downstream of the termination codon (Nicholson et al., 2010). These genes are also part of the process of SRP-dependent co-translational protein destined for the endoplasmic reticulum.

It is known that these proteins are involved in translation particularly the gene EEF1A1, an isoform of the alpha subunit of the elongation factor-1 expressed in lungs and arteries, which is responsible for the GTP-dependent binding of aminoacyl-tRNA to the ribosome. Beyond its function in translation, EEF1A1 takes part in the innate immune system by activating directly the transcription of IFN-gamma (Maruyama et al., 2007). IFN-gamma activates immune cells, such as macrophages and natural killer cells, and stimulates the major histocompatibility complex (MHC) II-dependent presentation of antigens to the immune system (Schroder et al., 2004). This process could be downregulated due to a decrease in the translation rate of EEF1A1. Furthermore, B2M, another gene in the list of decreased translational rate, encodes one of the proteins that conform MHC class I found on the cell surface of all nucleated cells (Bernier, 1980). Together with TXNIP, RPS3A, and RPS27A, it is involved in the antigen processing and presentation. Moreover, this gene, along with S100A11, HSP90AB1, and also EEF1A1, regulates the exocytosis of granules containing inflammatory mediators in neutrophils (Lee et al., 2005). All these genes, with the exception of TXNIP and S100A11, are also highly expressed in the arterial tissue. We highlight the presence in our analysis of the HSP90AB1, a known chaperone that facilitates the maturation of a wide range of proteins and its attenuation has been related to idiopathic pulmonary fibrosis and cystic fibrosis (Haase and Fitze, 2015; Wang and Ni, 2016). Our analysis also shows a translation decrease in A2M, a plasmatic protein highly expressed in lung and artery that inhibits a broad spectrum of proteases, including trypsin, thrombin, and collagenase (Cater et al., 2019). A recent study confirms our prediction, A2M level in sera from COVID-19 patients is significantly lower than in healthy subjects (Shen et al., 2020). Furthermore, A2M has a key role in regulating inflammatory processes because is able to bind to proinflammatory ligands (Feige et al., 1996). This is particularly relevant in the context of the COVID-19-derived cytokine storm.

In addition, we have also identified two genes (SAT1 and MGP) belonging to the pathway of endothelial cell calcification regulated by NOTCH1 (White et al., 2015). This finding acquires special relevance in the context of the acute lung injury (ALI) observed in many infected patients (Huang et al., 2020). The first gene SAT1 catalyzes the acetylation of polyamines (spermidine and spermine) and carries it out of the cell. The polyamine excess is a prominent source of oxidative stress that can increase inflammatory response (Hussain et al., 2017). Polyamines have also been connected with the immune system (Pérez-Cano et al., 2003). On the other hand, the MGP gene encodes the matrix gla protein, which is also highly expressed in all vasculatures. Recent studies suggest that MGP downregulates the tissue calcification by sequestering bone morphogenetic proteins (White et al., 2015). Mutations in this gene cause Keutel syndrome, which is characterized by peripheral pulmonary stenosis, abnormal cartilage calcification, and skin rashes (Munroe et al., 1999).

Another interesting protein predicted to be downregulated during the infection in lung and arterial tissues is SPARC-like 1 (SPARCL-1), also known as Hevin, commonly associated with regulation of cell migration and modulation of extracellular matrix proteins (Girard and Springer, 1996), and it has been shown to be involved in lymphocyte transendothelial migration through high endothelial venules (Girard and Springer, 1995). In this context, it is important to mention that clinical studies over patients that suffer several cases of COVID-19 document a dysregulation of immune response related particularly to a lower lymphocytes count (Huang et al., 2020). In addition, our analysis reveals a translational rate decay of fibronectin (FN1), a master organizer of extracellular matrices that mediates cellular interactions playing important roles in cell adhesion, hemostasis, and thrombosis (Pankov and Yamada, 2002; Wang and Ni, 2016). This prediction is in agreement with previous data showing that cells infected with SARS-CoV undergo downregulation in fibronectin expression (Surjit et al., 2004).

## 4. Discussion

The viral infection of human cells triggers an ensemble of host processes based on the interferons that interfere with viral replication. These processes have co-evolved with the viral response to the host defense, and the virus counteracts these processes through a diversity of immuno-modulatory mechanisms. For example, NS1 protein plays a central role in the influenza infection by suppressing the host IFNs response. Recent results suggest that NS1 protein can hamper the host gene expression at the translational level by obstructing the mRNA entrance tunnel of ribosomes (Thoms et al., 2020). Furthermore, the N protein of porcine reproductive and respiratory syndrome virus impairs the IFN transcription by acting over the TXK, which together with the EEF1A1 and PARP1 form the trimolecular complex that binds to the IFN-γ gene promoter (Maruyama et al., 2007; Kenney and Meng, 2015). Hemagglutinin of IAVs has been shown to facilitate IFNAR ubiquitination and degradation, reducing the levels of IFNAR, and thus suppressing the expression of IFN-stimulated antiviral proteins (Xia et al., 2016). These examples illustrate the action of viral dedicated factors that downregulate the transcription of IFNs. Up to the present, no dedicated factor with analog function has been identified in SARS-CoV and SARS-CoV-2. However, a recent report found a significant lack of IFN type I and III at the transcriptional level in human alveolar adenocarcinoma cells (Blanco-Melo et al., 2020). On the other hand, a marked upregulation of inflammatory mediators at the protein level (CXCL10, CCL2, IFN-α, and γ) has been observed in patients with SARS-CoV without a significant amount of specific antibodies (Cameron et al., 2007). Several cases of absence of protective immunity due to previous infection seem to indicate a similar landscape for COVID-19. Until now, the manner in which some patients fail to develop adaptive immunity is yet to be elucidated. As mentioned in the Results section, the decreased translation of B2M could be related to this last observation since is a crucial factor for the stable presentation of antigens derived from virus or tumor proteins; these antigens are recognized by cytotoxic T cells that eventually eliminate the target cell stimulating apoptosis to prevent systemic dissemination of the disease (Hulpke and Tampé, 2013).

MGP is also expressed at high levels in heart, kidney, and lung which is particularly interesting in the context of several comorbidities and collateral effects observed in the COVID-19 patients (Nikolich-Zugich et al., 2020). For example, skin rashes were recently reported as a new symptom of COVID-19 and the authors postulate that recognizing rashes is important to identify new and earlier COVID-19, cases (Bataille et al., 2020). In this context, we highlight that permanent skin rashes were reported as a characteristic of the Keutel syndrome, and mutations in the MGP gene were reported as a crucial factor in this syndrome (Munroe et al., 1999; Khosroshahi et al., 2014). In this manner, our results are related to these observations.

Summing up, if the depletion of a selected set of tRNA, induced by virus replication, affects the expression level or the co-translation folding of these proteins, one could expect the emergence of several systemic disorders.

## 5. Conclusion

Codon usage bias is thought to have significant effects on translation rate, where rare codons are assumed to be translated more slowly than common codons (Piovesan et al., 2013). It is assumed that rare and common codons are defined by usage rates of highly expressed genes. However, whether the codon composition of viral ORFome can affect the translation rate of host genes has not been thoroughly explored yet. Here, we have shown that the synthesis of the proteins associated with highly expressed genes, and with similar codon usage to the one of the virus, appears to be downregulated. This finding is in agreement with recent observations in totivirus-infected yeast (Chen et al., 2020). Following this idea, we determined which genes in lung could be affected by the viral replication. A functional analysis of these genes reveals that they could be related to collateral effects observed in COVID-19 patients (Huang et al., 2020). Further studies are mandatory to corroborate or discard the putative relationship established here.

One of the main obstacles in the recent development of vaccines has been the finding of increased infectivity observed to occur after immunizations with whole virus vaccines or complete spike protein vaccines. This phenomenon has been observed both in vaccines against SARS coronavirus and in respiratory syncytial virus. However, just as the virus regulates the translation of the host by its codon usage, the biotechnological manipulation of the frequency of codons could be used to design attenuated viruses. In this sense, other vaccine strategy has been recently assayed, focusing on altering the codon-pair usage without affecting protein sequence. This codon deoptimization strategy has reduced virus replication (Coleman et al., 2008; Le Nouen et al., 2014). We believe that our results shed light on how codon use could affect virus attenuation and would help decrease the damaging side effect, providing an exciting opportunity for live-attenuated vaccine development.

## Data Availability Statement

Publicly available datasets were analyzed in this study. This data can be found here: http://corona.papers.biochem2.com; http://proteomecentral.proteomexchange.org/cgi/GetDataset?ID=PXD017710.

## Author Contributions

LD designed the experiments. AA prepared the data. LD and AA the analyzed the data and wrote the manuscript. All authors contributed to the article and approved the submitted version.

## Conflict of Interest

The authors declare that the research was conducted in the absence of any commercial or financial relationships that could be construed as a potential conflict of interest.
